# A new computational approach to estimate whole-brain effective connectivity from functional and structural MRI, applied to language development

**DOI:** 10.1038/s41598-019-44909-6

**Published:** 2019-06-11

**Authors:** Gerald Hahn, Michael A. Skeide, Dante Mantini, Marco Ganzetti, Alain Destexhe, Angela D. Friederici, Gustavo Deco

**Affiliations:** 1 Center for Brain and Cognition, Computational Neuroscience Group, Department of Information and Communication Technologies, Universitat Pompeu Fabra, 08018 Barcelona, Spain; 2 Unit for Neurosciences, Information and Complexity (UNIC), CNRS, 91190, Gif-sur-Yvette, France and The European Institute for Theoretical Neuroscience (EITN), 75012 Paris, France; 3 Department of Neuropsychology, Max Planck Institute for Human Cognitive and Brain Sciences, 04103 Leipzig, Germany; 4 Research Center for Motor Control and Neuroplasticity, KU Leuven, 3001 Leuven, Belgium; 5 Functional Neuroimaging Laboratory, Fondazione Ospedale San Camillo - IRCCS, 30126 Venezia, Italy; 6 Institució Catalana de la Recerca i Estudis Avançats,Universitat Pompeu Fabra, 08010 Barcelona, Spain

**Keywords:** Reading, Network models

## Abstract

Recently introduced effective connectivity methods allow for the *in-vivo* investigation of large-scale functional interactions between brain regions. However, dynamic causal modeling, the most widely used technique to date, typically captures only a few predefined regions of interest. In this study, we present an alternative computational approach to infer effective connectivity within the entire connectome and show its performance on a developmental cohort with emerging language capacities. The novel approach provides new opportunities to quantify effective connectivity changes in the human brain.

## Introduction

The cortex hosts a variable number of structurally and functionally different areas that contain neurons and synapses, the building blocks of cortical computation^1^. Their electromagnetic activity can be picked up directly by methods like EEG or MEG and indirectly by measurements of blood flow changes using functional MRI^2^. This activity can show various degrees of statistical dependence, i.e. functional connectivity (FC)^3,4^ and forms entire networks of functionally connected cortical regions during task conditions and spontaneous activity^5–9^.

Cortical areas are connected through an extensive web of axonal fibers that can be traced and quantified with invasive^10^ and modern non-invasive techniques such as DTI^11^. This anatomical network is generally referred to as structural connectivity (SC). It provides the substrate for communication between spatially distant areas^12^ and integration of functionally segregated information across the cortex^13^. It was previously shown that such networks of anatomical connections can predict the strength of functional connectivity^14–18^.

In contrast, how strongly a stimulus propagates in cortical networks and causally influences neuronal activity in other areas is governed by the abstract notion of effective weights, which represents the time dependent efficacy of interactions between brain regions (effective connectivity, EC). These weights incorporate structural information related more to the density and strength of synapses rather than axonal fiber bundles, and they are also determined by the excitability of each brain region^3^. These effective connections directly determine correlations between anatomically linked areas and indirectly through transitive interactions and network effects due to global brain signals, i.e. synchronization of neuronal activity and BOLD signals across large parts of the brain^19,20^. Currently, several methods exist that allow the indirect estimation of directed effective weights and causal interactions. To date, the most widely used approach for estimating EC is dynamic causal modeling (DCM), in which neural activity in each brain area is approximated by mathematical models^21^. In general, DCM evaluates effective connectivity parameters for a number of different connectivity patterns, which are pre-specified by the modeler, and selects the best model based on a tradeoff between data fit and model complexity using a Bayesian approach. Even though DCM has been successfully applied to brain activity datasets recorded with various techniques and under different conditions, the scope of the EC estimation is usually limited to a few brain areas^22–25^, even though attempts to expand DCM to the whole brain have been made recently^26,27^. Notably, limiting EC estimation to only a few brain areas may give spurious results, since the effective interaction between two regions depends on their overall activation, which is not just a function of a few regions of interest, but is probably influenced by distributed processing across the entire brain^28^.

In this study, we present an alternative approach to approximate effective connectivity based on the dynamics of a whole-brain computational model which gradually infers undirected EC through a gradient descent algorithm by minimizing the distance between model and empirical functional connectivity. This technique derives effective weights of connections given by the white-matter-structural connectivity. Structural connections of 66 cortical regions^11^ were measured in 65 participants from four different age groups (3–4, 6–7, 9–10-year-old children and adults) using diffusion-weighted MRI. Empirical functional connectivity maps were constructed based on the Pearson correlation coefficient of the BOLD time series, recorded while individuals were performing a sentence comprehension task.

We apply this new method to study the development of effective connections at the whole brain level. All three connectivity types (SC, EC and FC) are reconfigured as the maturation of white matter tracts progresses from childhood to adulthood^29^ and elaborate cognitive abilities develop^30–33^. These changes are reflected in significant age differences within individual connections^34^ or graph theoretical measures^35^. Here, we test to what extent functional connectivity measured at different developmental stages is explained by the dynamics of a nonlinear whole brain model that is either wired according to empirically measured structural connections or computationally determined effective links. Furthermore, we investigate how a subset of two core cortical areas involved in language processing change their effective interaction during development.

## Results

**Figure 1:**
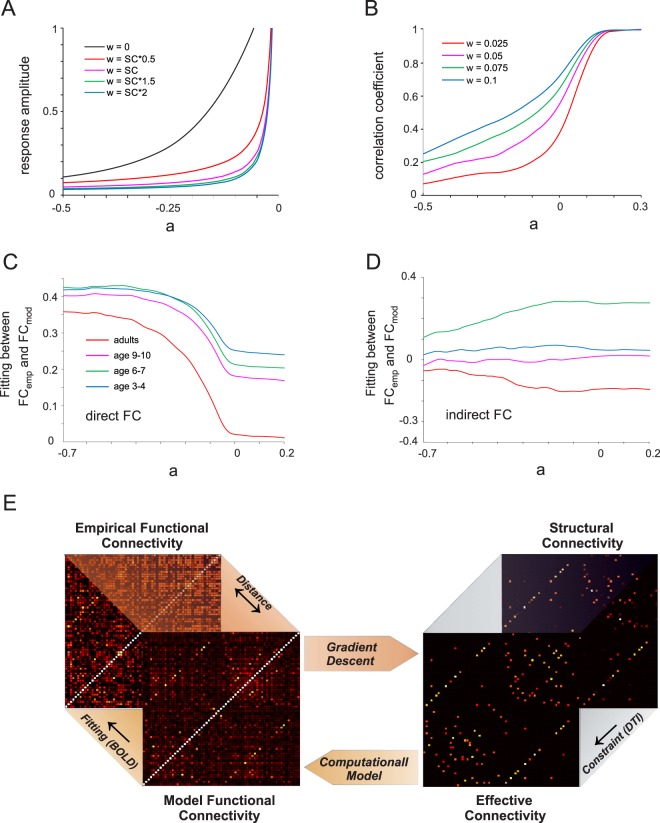
Predicting empirical FC from local node dynamics and principles of effective connectivity estimation. (**A**) Mean response amplitude defined as the power spectrum peak amplitude across all 66 Hopf nodes of a network wired according to empirical structural connectivity of the adult age group and as a function of the bifurcation parameter (a). The SC matrix was scaled by different values of the global coupling parameter (G) with zero indicating an unconnected network. (**B**) Mean correlation coefficient between the time series of all model nodes as a function of the bifurcation parameter (a) and the global coupling parameter (*G*). The bifurcation parameter was set and varied equally for all nodes. (**C**,**D**) Fitting (correlation coefficient) between empirical and model FC calculated for different values of the bifurcation parameter (a) and split into direct FC (**C**) and indirect FC (**D**). The global coupling parameter (G) was kept constant. (**E**) Working principles of effective connectivity estimation. Structural connectivity measured by DTI provides the anatomical pattern of connections which is used as a seed for the effective connectivity estimation. The model optimizes the fit between the functional connectivity computed from the model and the functional connectivity estimated empirically from BOLD time series. The distance between both functional connectivity matrices is used to update the effective connectivity matrix using a gradient descent approach. This procedure is repeated until the estimated EC remains stable.

The aim of this study was to explain empirically found BOLD signal correlations (FC) by effective connectivity between brain areas that was estimated with a whole brain computational model. BOLD signals were recorded from four different age groups and parcelleated into 66 areas according to the Hagmann scheme^11^. Signal correlations were assessed with the Pearson correlation coefficient. To model effective interactions, we employed a nonlinear mathematical model based on the normal form of a supercritical Hopf bifurcation, which mirrors the oscillatory nature of BOLD signals and incorporates a bifurcation parameter (a) that controls the responsiveness of a model node to input (Fig. 1A) and the interaction strength between different model nodes (Fig. 1B). This model was selected as it represents an approximation of the dynamics of excitatory-inhibitory spiking networks^36^, which undergo a similar bifurcation between noise-induced and self-sustained oscillations. It recently succeeded in reproducing statistics of EEG^37^, MEG^38^ and fMRI signals^39^. We did not explicitly incorporate a hemodynamic response function (HRF) that converts neuronal model activity into BOLD signals, since recent modeling efforts show that the model FC remains largely preserved after addition of an HRF^40^. Similar to the empirical data, we modeled the activity of 66 areas with the Hopf formalism and connected the nodes based on the structural information obtained for each group via diffusion MRI. This information was parcellated according to the Hagmann matrix^11^ and contained the pattern of connectivity together with connection strength represented by axonal fiber density, which serves as a proxy to the real synaptic connectivity weights.

Before estimating effective connectivity, we tested how much of the empirical FC can be already explained by the dynamical properties of the model, as studied previously^41^. We thus systematically varied the bifurcation parameter uniformly across all model areas and estimated functional connectivity between modeled brain areas (FC_mod_) and their similarity with empirical functional connectivity (FC_emp_) by calculating the Pearson correlation coefficient between both matrices. Both direct and indirect functional connectivity were calculated, which reflect activity correlations between areas with or without an existing structural connection, respectively. The results show that direct FC similarity between simulated and experimental data increased as the bifurcation parameter became more negative, with a maximum value of ~0.35 for adults and 0.4–0.42 for children (Fig. 1C). Except for the age group 6–7, indirect FC showed no correspondence between model data and recordings (Fig. 1D) for any value of a. When the bifurcation parameter was adjusted for each node separately (see Materials and Methods), the fit for direct FC slightly decreased, while it remained unchanged for indirect FC (Supplementary Fig. 1A,B). These findings illustrate that variations of local model node dynamics are insufficient to fully capture the functional connectivity in the recorded data.

### Estimation of effective connectivity

Next, we modeled effective connectivity which represents the strength of activity flow between brain areas. In order to find effective weights, we applied a gradient descent approach that optimizes the correspondence between empirical and model FC. The main principle of this approach is illustrated in Fig. 1E. As activity only propagates between areas that are physically connected, we based the computation of effective connections on the presence of a link in the SC matrix. EC estimation followed a gradient descent algorithm that adapts the weights of the structural connectivity to maximize the similarity between the modeled FC and empirical FC. We refer to the resulting matrix as EC, which has the same patterns of connections as the original SC, but differs from it with respect to the connectivity weights. First, we simulated the FC_mod_ based on the structural connections given by the SC matrix. The FC_mod_ and FC_emp_ were then compared by computing the Pearson correlation coefficient between both matrices. Subsequently, we updated the original structural matrix according to the following rule:

1 $${\rm{\Delta }}E{C}_{(i,j)}=\varepsilon \,(F{C}_{emp(i,j)}-F{C}_{mod(i,j)})$$

where ε is a learning constant set to varying values in this study. This gradient descent algorithm was repeated and after each optimization step the connections of the EC matrix were rescaled repeatedly by a global dynamics parameter (Fano factor, see Materials and Methods, Supplementary Fig. 2 and Supplementary Discussion). This guaranteed that the global dynamics in the simulation remained comparable to the empirical data throughout the optimization process. The optimization was stopped after 300 steps when the EC matrix had in general already stabilized, as determined by a convergence of the correlation between SC and EC matrices to a stable value. To validate that the gradient descent can correctly recuperate the underlying effective interactions on the basis of a given SC and FC_emp_, we applied the method to ground truth data with a-priori known EC (Materials and Methods). This approach was indeed able to recover the EC with an accuracy up to 0.98 for both direct and indirect EC (Supplementary Fig. 3A–D and Supplementary Discussion).

### Empirical effective connectivity

**Figure 2:**
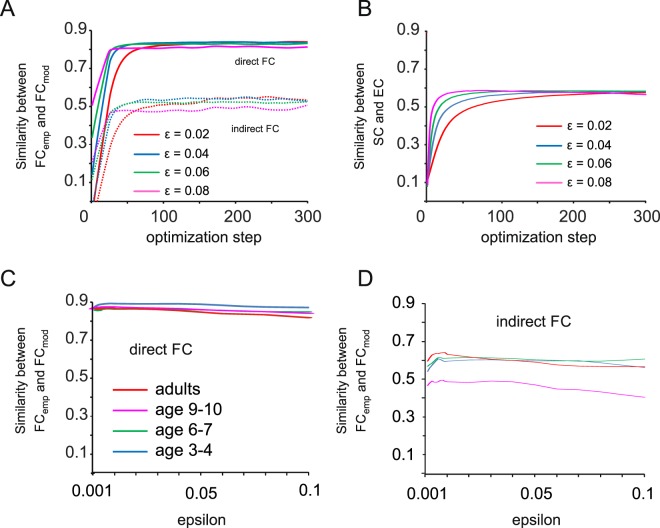
Estimation of effective connectivity from empirical data. (**A**) Pearson correlation coefficient (similarity) between FC_emp_ and FC_mod_ separated into direct and indirect FC as a function of gradient descent optimization steps. Traces are shown for different values of the gradient descent learning parameter ε and are based on optimization of the adult group dataset. (**B**) Same as in (**A**) for the correlation coefficient between SC and EC. (**C**) Correlation coefficient (similarity) between empirical and model FC of direct connections for all age groups shown for different values of ε. (**D**) Same as in (**C**) for indirect functional connections.

We applied this approach to obtain effective connectivity from each age group based on the structural connectivity pattern. An example of direct and indirect FC estimation during the optimization process representing the adult group is shown in Fig. 2A for different values of the learning parameter ε. Interestingly, the correlation between SC and EC, our measure for a stable convergence to a particular effective connectivity pattern, converged later during the optimization procedure (Fig. 2B). A more detailed analysis of the performance of the algorithm as a function of ε revealed a peak of direct FC optimization at ε ~ 0.01 with a fit of ~0.85 for all age groups except for the youngest children with a fit close to 0.9 (Fig. 2C). For indirect FC, a maximum was found for the same ε value and an FC fit of ~0.6 for three age groups with children aged 9–10 showing a lower fit of ~0.5 (Fig. 2D). To test whether the EC is reliably estimated, we repeated the recovery process 25 times per age group with different initial conditions by randomly altering the original weights of the SC matrix, while keeping the pattern of connected and unconnected areas unchanged. The effective connectivity across all trials showed a correlation of ~0.99, providing strong evidence that the EC estimate is reliable and independent of initial conditions. Furthermore, we examined the possibility of overfitting, i.e. that the derived EC (N = 2178 fitted connections) is only valid for the fitted dataset and does not generalize to novel data. To this end, we repeatedly split the empirical data of each age group into training and test datasets (half-half), each time with a different set of subjects. We then calculated the EC for the training dataset and computed the correlation coefficient between the FC_emp_ matrix in the test dataset and the FC_mod_ matrix that was obtained from simulations either from the computed training set EC matrix or the SC matrix. The average FC_emp_ – FC_mod_ correlation using the training set EC as the basis for the FC_mod_ calculation (adults: 0.56 ± 0.04; age 9–10: 0.56 ± 0.04; age 6–7: 0.42 ± 0.04; age 3–4: 0.41 ± 0.05) remained indeed significantly higher for each age group than the average FC_emp_ – FC_mod_ correlation based on simulations with the SC (adults: 0.3 ± 0.02; age 9–10: 0.36 ± 0.01; age 6–7: 0.37 ± 0.03; age 3–4: 0.36 ± 0.02, t-test: p < 0.0001 for all four group comparisons). Thus, despite its complexity, the EC model generalizes better to the data than the SC model.

### Effective node strength

**Figure 3:**
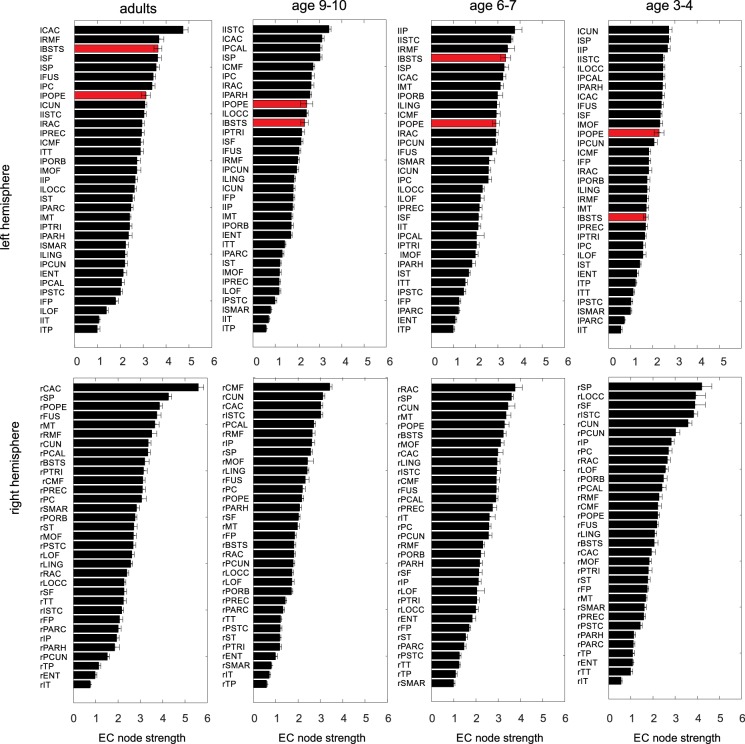
Effective node strength (Mean + SD) for all cortical areas of the Hagmann parcellation, age groups and both hemispheres, ordered from largest to smallest values. Red: two core areas implicated in language processing.

To compare EC across age groups, we used the graph theoretical measure of weighted node strength, which quantifies the overall strength of all connections impinging on a given area. The effective node strength for each area, hemisphere and age group, sorted from highest to lowest values, is shown in Fig. 3. The numbers reflect the mean node strength across 20 simulations that were based on a jack-knife approach (see Materials and Methods).

We then examined how information stored in the EC node strength differs from the empirical FC node strength. To this end, we calculated the Pearson correlation coefficient between the strength of all FC_emp_ and the EC nodes for all simulations and found a value of 0.61 for adults, 0.64 for age 9–10 and age 6–7, and 0.8 for age 3–4. In comparison, the correlation between FC_emp_ and SC node strength, revealed values of 0.07 for adults, 0.31 for age 9–10, 0.2 for age 6–7 and 0.33 for age 3–4. These results suggest that both SC and EC remain rather dissimilar to the empirical FC.

**Figure 4:**
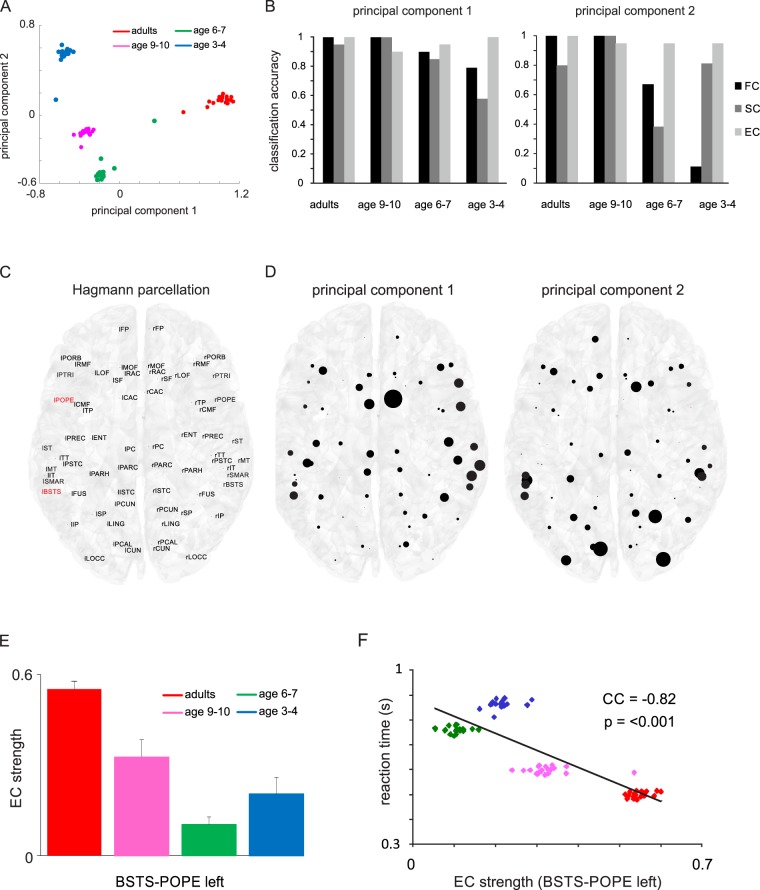
Effective node strength differences between age groups. (**A**) Two- dimensional principal component space of EC node strength across all 66 areas for all age groups. Each data point was obtained by PCA of EC node strengths from all subjects of a group leaving one subject per group out. (**B**) Classification accuracy of age groups using a naïve Bayesian classifier based on the first two PCs of the FC, SC and EC node strength. (**C**) Hagmann parcellation scheme with abbreviated names of cortical areas. Two core regions involved in language processing are highlighted in red. (**D**) Coefficients of first two principal components of EC node strength projected onto the Hagmann parcellation scheme. The diameter of each circle represents the variance of EC strength for a given area across different age groups. (**E**) EC strength histograms of the connection between two language areas. Error bars: SD. (**F**) Correlation between reaction times during the language task and EC weight of the two language areas paneled for the different age groups.

To quantify age differences in node strength and compare across the three connectivity types, we reduced FC, SC and EC information of all 66 areas and 20 simulation per age group to its first two principal components (see Fig. 4A for EC and Supplementary Fig. 4A,B for FC and SC). The appearance of distinct clusters in the PCA plane illustrates that information in the EC at the population level can readily distinguish between age groups. The separation of clusters is less prominent in the FC and SC for the two age groups of the youngest children. To further test the power of the first two PCs to separate age groups using FC, SC and EC, we applied a naïve Bayesian classifier (Fig. 4B). The results show that adults and children age 9–10 can be classified by all connectivity types with (or close to) 100% accuracy using either the first or second PC, with the exception of SC which has a reduced adult classification performance (0.8) for the second PC. Notably, classification performance for the two youngest children groups is largely reduced for FC and SC in both principal components, but remains close to 100% for the EC. These results demonstrate that effective connectivity can surpass structural and functional connectivity in extracting age-related information.

Thereafter, we tested the individual contributions of cortical areas in the Hagmann parcellation (Fig. 4C) to either the first or second principal component of the EC node strength. The size of the PC coefficients for both PCs is shown in Fig. 4D, represented by the diameter of the filled circles. For the first PC, which revealed large age-related variance between adults and children, the anterior and posterior cingulate cortex, show the largest age differences in the EC node strength. The second PC relates to large age variance in the lateral occipital cortex, the superior parietal cortex and the cuneus.

### Effective connectivity of the language network

Finally, after evaluating age-related changes in effective connectivity at the whole-brain level, we applied this approach to the language network. In our previous studies, we have demonstrated that the cognitive refinement of language comprehension skills is closely linked to the gradual functional specialization of the left inferior frontal and posterior superior temporal cortex^42^, as well as the maturation of the interconnecting white matter fiber tract (i.e. the left arcuate fasciculus)^33^ that forms the structural basis of the uniquely human language faculty^43^. Accordingly, we focused on age-related changes in the effective interaction of these two areas. Effective connectivity differed significantly between all subgroups (ANOVA: p < 0.0001; all family-wise error-corrected post hoc t-tests: p < 0.0001) with a decrease between age 3–4 and 6–7 and strong increases at age 9–10 and in adulthood (Fig. 4E). Moreover, the age difference in EC was correlated with language comprehension as measured by reaction times during the language task (Fig. 4F).

## Discussion

In this study, we applied a new computational model to study the origin of whole brain functional connectivity during a language task performed by four different age groups. This model describes a supercritical Hopf bifurcation and can reproduce the behavior of interacting excitatory and inhibitory neurons, which ranges from constant firing to damped and sustained oscillations^36,44,45^. We found that the changes in the local dynamics of the model were able to predict direct empirical FC with accuracy of ~0.4, while indirect FC remained poorly explained. This finding is in accordance with previous studies of resting state dynamics which have shown that anatomically more strongly connected areas also display stronger activity correlations^46^. Moreover, the discrepancy between direct and indirect FC prediction is also in line with recent results from a comprehensive theoretical study using a variety of models to predict empirical FC^47^. This limited predictive power might be explained by the nature of the structural information from DTI data which may provide an accurate pattern of the structural graph in the brain, but may reveal connection weights (estimated as axonal fiber densities) that are too inaccurate to capture the true synaptic interaction strength between areas. In addition, synaptic weights are subject to short-term plasticity^48^, which may alter the interaction strength especially during a task setting.

To overcome the lack of experimental knowledge about neuronal interaction parameters, we resorted to the concept of effective connectivity which is defined as the causal interaction between two areas and is equivalent to the response magnitude in one area to a given input from another area^4,49,50^. This response is a function of local network dynamics and synaptic weights, and the influence of both is reflected in the effective interaction strength. As effective connectivity captures the biophysical transfer of activity between brain areas along structural pathways, it fundamentally differs from functional connectivity which only manifests the measurable consequences of such interactions, described as correlations. Importantly, biophysical interactions may not be adequately represented by correlations, as they are also elevated by other mechanisms such as common input to structurally unconnected areas and global synchronization. Moreover, since the degree of activity transfer between brain areas cannot be inferred experimentally, a neuronal model is usually specified to infer effective connectivity weights. Once the effective interaction graph of a network is known, activity from model simulations that incorporate effective coupling strength between brain areas, by definition, fully captures functional connectivity between connected and unconnected regions^49^.

In this study, we used a novel approach to estimate effective connectivity at the whole brain level from empirical functional and structural data. We showed that our model together with a simple gradient descent algorithm^40^ can recuperate both direct and indirect FC as well as effective connectivity in ground truth simulations with high fidelity. When applied to the language task fMRI recordings, the empirical direct FC was explained up to a value of ~0.9, while explanatory power for indirect FC was somewhat weaker. As we showed, insufficient duration of the empirical dataset can lead to reduced predictive power of our model and may also account for a reduced fit of indirect FC as compared to direct FC (see Supplementary Material). Correlations between areas that are not directly connected are partly generated by network effects and global synchronization^19,20,51^ which need sufficient recording and simulation time to be captured accurately by the model. Another possibility reflects our restriction to symmetric interactions, as indirect FC may also be shaped by the asymmetry of the underlying structural links^40^.

This approach represents a whole brain alternative to dynamic causal modeling which is the current gold standard in effective connectivity modeling. In contrast, DCM is usually restricted to EC estimation of a few regions of interest in the brain, with recent efforts to make DCM amenable to the whole-brain level^26^. An important difference between these two methodologies is that the gradient descent is currently restricted to undirected interaction strength between two areas, while DCM also takes directed effects into account. However, a recent study has successfully inferred directed EC weights from time-shifted functional connectivity data by applying a gradient descent algorithm similar to this study, but using a simplified Ornstein-Uhlenbeck noise process to model BOLD activity^40^. Extracting directed EC with the model used in our study remains a future avenue of research.

While the development of effective connectivity has been investigated in various contexts using DCM^52^, we applied our model to compute effective connectivity within the entire connectome to detect developmental changes in information transfer during a specific language processing task. Our classification results show that EC reflecting a combination of SC and FC information can help to extract age-related information which is more difficult to obtain using structural or functional data alone^53^. Our approach also offers full flexibility to focus on a subset of predefined areas such as language specific regions. Here, we provide evidence for the feasibility and validity of such an analysis by showing that effective connectivity within the core language network increases towards adulthood. This finding complements our previous studies indicating a gradual increase in functional selectivity and white matter maturity in this system^33,42^. The lower effective connectivity in 6–7-year olds compared to 3–4-year olds in our current work should be noted as a discrepancy with respect to our previous work. It might be explained by the differences in ROI selection or by the present modeling assumption that information is symmetric and not under hierarchical control of the inferior frontal cortex. In addition to the language system, we also observed pronounced changes in effective connectivity from childhood to adulthood in other brain networks, especially along midline structures belonging to the brain’s rich club^54^. As these rich club nodes are well connected among themselves and other brain areas, their maturation may be associated with enhanced large-scale information transfer and possibly better task performance^55^.

## Conclusion

In summary, we derived effective connectivity at the whole-brain level based on structural data and functional connectivity measured with fMRI. To this end, we employed a novel computational model and a simple gradient descent algorithm on language task data. We were able to unravel age differences in causal interactions between specific areas of interest related to language processing and other brain networks. Our results supplement previous anatomical and functional studies on the development of language skills and may provide important insights into general principles of brain development in the future.

## Materials and Methods

### Participants

Functional MRI data were available for 80 healthy human volunteers from 4 different age groups: 20 children from 3 to 4 years of age (y.o.a.) (mean: 4;4, range: 3.9–4.11 y.o.a.), 20 children from 6 to 7 y.o.a. (11 females, mean: 7.5; range: 6.7–7.11 y.o.a.), 20 children from 9 to 10 y.o.a. (8 females, mean: 10.3, range: 9.7–10.11 y.o.a.) and 20 adults (7 females, mean: 26.5, range: 21.8–33.6 y.o.a.). Diffusion-weighted MRI data were available for 65 participants comprising 12 datasets from the 3- to 4-year-old children (8 females, mean: 4.7, range: 3.10–4.11 y.o.a.), 17 datasets from the 6- to 7-year-old children (9 females, mean: 7.3, range: 9.9–7.11 y.o.a.), 17 datasets from the 9- to 10-year-old children (7 females, mean: 7.6, range: 6; 7–7.11 y.o.a.), and 19 datasets from the adult participants (7 females, mean: 26.4, range: 21.9–33.8 y.o.a.). The adult participants and parents/legal guardians of the children gave voluntary written informed consent and the children agreed verbally to participate in the experiment which was approved by the University of Leipzig Ethical Review Board. Detailed inclusion criteria as well as demographic and psychometric data are described elsewhere^33^. The applied methods were performed in accordance with the relevant guidelines and regulations.

### Behavioral task

The fMRI experiment consisted of 96 target trials and lasted ~15 minutes. During the behavioral language comprehension task, two pictures and spoken sentences were presented to the participants, who were asked to match the meaning of the heard sentence with the corresponding scene in one of the two pictures via a button press. Participants were instructed to attend to the pictures, listen carefully to the sentences, and respond as fast as possible as soon as auditory stimulation was over. Details regarding task, stimuli and the experimental procedure can be found elsewhere^42^. To test whether reaction times to the task correlate with the development of EC, we created N (i.e. number of subjects within a group) datasets for each age group by averaging the reaction times of N-1 subjects, each time removing one subjects from the averaging process.

### Functional MRI data: acquisition, preprocessing and connectivity matrix computation

Functional MRI data were acquired on a 3.0-Tesla Siemens TIM Trio (Siemens AG) whole-body magnetic resonance scanner using a 12-radiofrequency-channel head coil. A BOLD-sensitive T2*-weighted gradient-echo echo-planar imaging (EPI) sequence was applied to 26 slices with TR = 2 s, TE = 30 ms, FOV = 192 mm, matrix size = 64 × 64 voxels and voxel size 3 × 3 × 3 mm³. In order to correct for geometric distortions in EPI caused by magnetic field inhomogeneity, a field map was obtained for each dataset. For anatomical localization, T1-weighted three-dimensional magnetization-prepared rapid-acquisition gradient echo (MPRAGE) pulse sequences with TR = 1.480 ms, TE = 3.46 ms, TI = 740 ms, FOV = 256 × 240, matrix size = 256 × 240 × 128 and voxel size = 1 × 1 × 1.5 mm³ were acquired.

Functional images were preprocessed using SPM8 (http://www.fil.ion.ucl.ac.uk/spm/software/spm8/). First, a cubic spline interpolation algorithm was applied to the time series of individual slices to correct for time differences between slices recorded within the same scan and to resample them afterwards (slice time correction). Second, images were realigned (i.e., spatially registered and transformed) to the first acquired image to correct for movement between scans, and then unwarped to correct for distortions caused by magnetic field inhomogeneties and interpolation artifacts (motion correction). Third, the head motion parameters and their first derivatives as well as white matter and cerebrospinal fluid signals and their first derivatives were regressed out (nuisance regression). Fourth, low-resolution functional images of each participant were mapped (i.e., coregistered) onto the corresponding high-resolution T1-weighted structural images and subsequently normalized to a Montreal Neurological Institute (MNI) template covering the whole age range^56^ (spatial normalization) in order to provide a common space for the group comparisons. We ensured that the scaling did not affect the functional data by also normalizing them to MNI templates exactly matching the group-specific mean age and afterwards comparing both volumes against each other. This did not reveal any significant differences. Finally, data were spatially low-pass filtered using a 4 mm full width half-maximum (FWHM) Gaussian kernel. The time series were subsequently temporally filtered between 0.01 and 0.04 Hz to reduce analysis to a peak frequency seen in the unfiltered data and allow for comparison with the results from the computational model which produced time series within a limited frequency band. However, such a narrow filter bandwidth may distort the correlation between the time series in our data and give wrong FC results^57^. To test for possible distortions, we also filtered the data in a more conventional frequency band (0.01–0.1 Hz) and compared the resulting FC matrix with the FC form the narrowly filtered data by computing the Pearson correlation coefficient. Importantly, the correlation coefficient was ~0.99 for all age groups, demonstrating that the correlation structure remains intact within this narrow low frequency band.

BOLD time series were calculated on averaged signal intensities in 66 regions of interest defined by T1-image-based parcellations of cortical regions with clear anatomical landmarks^11^. The BOLD signals of all subjects belonging to a given age group were concatenated to a single group signal to allow for a more reliable estimation of effective connectivity (see Results section). Functional connectivity matrices were created by calculating Pearson correlation coefficients between the concatenated BOLD signal intensity time courses of each region-of-interest pair. The group functional connectivity matrices were *z*-transformed applying Fisher’s *r*-to-*z*-transformation. We note that FC matrices obtained after concatenation of single subject data were almost identical to matrices calculated for each subject separately and subsequent averaging, as evaluated by their Pearson correlation coefficient (0.96–0.98 across all age groups).

### Diffusion-weighted MRI data: acquisition, preprocessing and connectivity matrix computation

Diffusion-weighted MR data were collected with the same hardware as the functional MR images using a twice-refocused spin EPI sequence with TE = 100 ms, TR = 9300 ms, matrix size = 128 × 128 voxels, voxel size = 1.7 × 1.7 × 1.7 mm³, 65 axial slices covering the whole brain. We used 60 isotropically distributed diffusion-encoding gradient directions with a b-value = 1000 s/mm². 7 anatomical reference b0 images without diffusion weighting were acquired once at the beginning of the sequence and after each block of 10 diffusion-weighted images for off-line motion correction. Fat saturation was applied together with 6/8 partial Fourier imaging and generalized auto-calibrating partially parallel acquisitions (GRAPPA) with acceleration factor = 2.

Diffusion-weighted images were preprocessed using FSL (http://www.fmrib.ox.ac.uk/fsl). Head motion correction was carried out using the 14 images without diffusion-weighting (b0) to estimate the motion correction parameters applying a rigid-body registration algorithm. Residual head-motion-corrupted image directions (max. 5) were removed from each dataset and the remaining images were corrected for eddy current distortions. Afterwards, the diffusion-weighted images were coregistered to the corresponding skull-stripped T1-weighted images in MNI space. Finally, a voxel-wise diffusion tensor was fitted to the datasets.

Whole-brain deterministic fiber tracking was carried out with the Camino Diffusion MRI Toolkit (http://www.cmic.cs.ucl.ac.uk/camino). The parcellations described above were spatially transformed into the individual space of each participant and slightly grown into the white matter. The regions including voxels at the grey-white matter interface were used as seeds for the tractography analysis. Subsequently, the total number of fibers, the fiber density and the average fractional anisotropy within each tract connecting each pair of seeds were calculated and structural connectivity matrices for each of the three indices were created. The simulations based on structural connectivity in the present study were based entirely on the fiber density, which was derived from the fiber count measure. The number of fibers between ROI A and ROI B was divided by the number of voxels at the surface of ROI A and of ROI B. The resulting number was called fiber density, and ranged between 0 and 1. Fiber density was equal to 0 when no fiber was connecting ROI A and ROI B. Fiber density was equal to 1 when fibers departing from each voxel at the surface of ROI A were arriving at each voxel at the surface of ROI B. The anatomical connectivity matrices were obtained for each subject and subsequently averaged across all subjects of each age group.

### Computational model

The computational model used in this study comprised N = 66 nodes, which was the same number of cortical areas extracted from the structural and fMRI data. The nodes were coupled by long-range inter-areal connections which were given by the structural connectivity matrix averaged across all subjects of each age group. The connection strength was equal to the fiber density of white matter tracts. Inter-areal conduction delays were not taken into account in the model. The activity of each neuronal node was approximated by the normal form of the supercritical Hopf bifurcation. This bifurcation describes a change from noise-induced damped oscillations to sustained oscillatory activity and is formalized by the following set of coupled equations:

2 $$\frac{{{\rm{dx}}}_{{\rm{j}}}}{{\rm{dt}}}=[{{\rm{a}}}_{{\rm{j}}}-{{\rm{x}}}_{{\rm{j}}}^{2}-{{\rm{y}}}_{{\rm{j}}}^{2}]{{\rm{x}}}_{{\rm{j}}}-{{\rm{\omega }}}_{{\rm{j}}}{{\rm{y}}}_{{\rm{j}}}+{\rm{G}}{\sum }_{{\rm{i}}}{{\rm{C}}}_{{\rm{ij}}}({{\rm{x}}}_{{\rm{i}}}-{{\rm{x}}}_{{\rm{j}}})+{{\rm{\beta }}{\rm{\eta }}}_{{\rm{j}}}({\rm{t}})$$

3 $$\frac{{{\rm{dy}}}_{{\rm{j}}}}{{\rm{dt}}}=[{{\rm{a}}}_{{\rm{j}}}-{{\rm{x}}}_{{\rm{j}}}^{2}-{{\rm{y}}}_{{\rm{j}}}^{2}]{{\rm{y}}}_{{\rm{j}}}+{{\rm{\omega }}}_{{\rm{j}}}{{\rm{x}}}_{{\rm{j}}}+{\rm{G}}{\sum }_{{\rm{i}}}{{\rm{C}}}_{{\rm{ij}}}({{\rm{y}}}_{{\rm{i}}}-{{\rm{y}}}_{{\rm{j}}})+{{\rm{\beta }}{\rm{\eta }}}_{{\rm{j}}}({\rm{t}})$$

The term $\beta {\eta }_{j}$ reflects additive Gaussian noise with standard deviation β = 0.01, and ω_j_ represents the intrinsic node frequency, which is related to the frequency *f*_*j*_ in the BOLD signal through the following expression: ${{\rm{f}}}_{{\rm{j}}}=\frac{{{\rm{\omega }}}_{{\rm{j}}}}{2{\rm{\pi }}}$. Node frequencies in the model were uniformly set to a value of 0.025* 2π, corresponding to the dominant frequencies seen in the empirical time series. The modeled data were subsequently filtered between 0.01 and 0.04 Hz to filter out higher frequencies generated by the noise and allow for an estimation of the model Fano factor distribution that can be compared with the empirical data. The bifurcation parameter a_j_ controls the dynamical behavior of the model which shows a supercritical Hopf bifurcation with a_j_ = 0. The bifurcation entails a stable fixed point with a_j_ < 0 with noise-driven oscillations, which is converted into a stable limit cycle with self-sustained oscillations with a_j_ > 0. Note, however, that the addition of connections to a network of Hopf nodes slightly shifts the bifurcation point away from zero to more negative values, as seen in a single unconnected node. The parameter G (global coupling parameter) scales each connection in the connectivity matrix (C_ij_) by a constant value. C_ij_ corresponds to either structural connectivity or represents effective connectivity, after repeated updating by the gradient descent EC algorithm. To compare empirical with model data, the variable x_j_ of each node *j* was used as a direct proxy for the BOLD signal, similar to previous studies^39,58,59^.

### Analysis of local dynamics

To analyze the excitability of a local Hopf node, i.e. its responsiveness to a given input, we fitted a 3^rd^ order polynomial to the power spectrum of the simulated time series of each area *i*. The peak of this polynomial *P*_*mod*(*i*)_ was taken as a measure for its responsiveness, and its relation with the bifurcation parameter as well as the global coupling parameter studied. We also attempted to adapt the local bifurcation parameters in the model such that the spectral peak in the model areas matched those found in the empirical data. To this end, we estimated empirical power spectrum peaks *P*_*emp*(*i*)_, similar to the model, for each area and rescaled the resulting values such that the sum of the power spectrum peaks in the recorded areas were equal to the model peaks that were found when the global model dynamics was in accordance with the empirical data (see section on global dynamics below). The bifurcation parameter of each area was then modified based on a gradient descent approach with the following equation:

4 $${\rm{\Delta }}{a}_{i}=\gamma \,({P}_{emp(i)}-{P}_{mod(i)})$$

where *γ* reflects the speed of convergence and was set to 1500 in this study. The update of a_j_ was repeated 100 times and after each step the similarity of spectral peak values in the model and the empirical data was assessed by computing the Pearson correlation coefficient between the two vectors.

### Analysis of global dynamics

To analyze global empirical and model dynamics which was used for subsequent estimation of effective connectivity we transformed the filtered time series into a point process by applying a threshold to the continuous data (Supplementary Fig. 2A, top^60^). Every peak in the time series that was found above the threshold, set to a value of zero in the z-scored data, was taken as an event with a time stamp given by the time of peak appearance. The discretized data were then binned with sliding windows of 5 TR, which was moved forward in steps of 1TR. Next, we summed up all events within each TR of a window, and subsequently computed the Fano factor (FF) of event counts across all five TRs within a window. The Fano factor was defined as:

5 $$FF=\frac{var(count)}{mean(count)}$$

For FF = 1, all events are uncorrelated and correspond to a Poisson process (Supplementary Fig. 2B). Global bursts of events result in higher order correlations and are characterized by a large variance in event counts due to a concentration of events within a few TR followed by TRs with absence of events. As a result, the Fano factor assumes values ≫ 1 and thus measures the degree of global synchronization of brain activity across all areas^61^. We obtained the distribution of Fano factors from all windows for each individual of an age group which were then merged into a single distribution for an entire age group. An exponential function with exponent λ was then fitted to both the model and empirical FF distributions. Deviations of the model FF distribution from the empirical distribution were assessed by computing the absolute difference between λ_emp_ and λ_mod_.

### Approximation of effective connectivity

The key aspects of the algorithm for estimating effective connectivity are described in the results section. Here, we provide more details of the simulation process. During EC estimation, we simulated model FC with an excitability value of *a* = −0.1, which was set uniformly across all areas. The resulting FC_mod_ was Fisher z-transformed and compared to the FC_emp_ by computing the Pearson correlation coefficient between the upper triangular parts of both matrices. After each optimization step, the connections of the EC matrix were rescaled repeatedly by $G=\frac{\langle F{F}_{emp}\rangle }{\langle F{F}_{mod}\rangle }$ until the absolute difference between λ of the exponential fit to the empirical and model FF distribution was < 0.15, such that the global dynamics in the simulation and the empirical data remained similar during the optimization procedure. For each age group, we conducted 25 simulations with different initial conditions that were generated by randomizing the SC weights to values between 0 〈 *w*_*i*_ 〉 1. We then rescaled the SC matrix with a constant term such that the sum of the randomized weights was equal to the sum of the weights in the original SC matrix. Note that no connections were added to the original SC matrix, except for links between homologous cortical areas that are known to be underestimated by modern DTI techniques^16^. When empirical functional connections were negative, the algorithm often yielded negative effective connections, in which case every update that led to negative weights was dismissed and the previous positive weight value kept. To test for overfitting, we split each age group into a training and test dataset each comprising N/2 subjects randomly chosen 100 times, except for children with age 3–4, where the training dataset included 10 subjects and the test set 9 subjects. We estimated the EC for each training set and compared the FC_mod_ that resulted from both the training set EC and the SC with the FC_emp_ of the test dataset by calculating the correlation coefficient between the empirical and model FC matrices.

### Model validation

To validate the model, we used the EC estimated from the adult group with the dynamical model as the ground truth EC based on which we generated an empirical FC with the Hopf model using a uniform value of *a* = −0.1 and setting G = 1. We then attempted to recuperate this FC with the effective connectivity algorithm described above, using 25 weight-randomized version of the original EC matrix (see previous section on SC randomization) as seeds. Optimization was performed using different simulation times of both the simulations used to generate the ground truth FC and its recovery.

### Graph theoretical analysis

Each EC matrix was quantified by calculating the weighted degree of each node, a graph theoretical measure that sums up the weights of all connections of a given area *i* with all other connections *j* ∈ *N*, where N is the set of all areas in the network. The node degree *k* is formally defined as:

6 $${k}_{i}^{w}={\sum }_{j\in N}{w}_{ij}$$

The resulting vector of 66 node degrees for each age group was used for further analysis.

### Age classification analysis

To classify EC matrices into their respective age groups, we first generated N (total number of subjects per age group) effective connectivity matrices, each time leaving out one subject and recuperating the empirical FC from the remaining N-1 subjects. The resulting EC node degree vectors from each dataset were used for a subsequent classification analysis. The same leave- one-out procedure was applied to the structural and empirical functional connectivity matrices to allow for classification performance across EC, SC and FC_emp._ Using principal component analysis, the high dimensional node degree information was then reduced to principal components (PC), which represented nodes with highly varying degree across all four age groups. The scores of the first two PCs were used as features for subsequent classification of participants with a naïve Bayesian classifier (NBC). Note that most age groups were represented by clearly separable clusters in this two-dimensional PC plane and we thus refrained in this study from using more formal measures to quantify cluster separation as used previously^62^. The NBC is a simple nonlinear classifier that is based on Bayes’ rule which predicts the class a participant belongs to by choosing the class with the highest posterior probability. This classifier requires class-conditional independency, which is given by using independent PCs as features. The estimated probability distribution for the predictors was assumed to be Gaussian in this study. The classification process was divided into a training phase and a prediction phase. During training, the classifier was fed with data using a leave-one-out cross validation strategy, in which all but one dataset of each age group were used to train the statistical model. The trained classifier was then used to predict the class of the remaining four datasets (one per group). Finally, classification accuracy was assessed by calculating the fraction of correct classifications after the cross-validation for each age category separately.

## Supplementary information

Supplementary Material

## Data Availability

The datasets analyzed during the current study are not publicly available due to constraints imposed by the ethics approval, but are available from the corresponding author upon reasonable request.
