# Supplementary material for: A new computational approach to estimate whole-brain effective connectivity from functional and structural MRI, applied to language development
Source: Sci Rep. 2019 Jun 11;9:8479. doi: 10.1038/s41598-019-44909-6 (PMC6559954; doi:10.1038/s41598-019-44909-6)
Supplement: Supplementary file 1 — Supplementary Material [file 41598_2019_44909_MOESM1_ESM.docx]

Supplementary Information

A new computational approach to estimate whole-brain effective connectivity from functional and structural MRI, applied to language development

Gerald Hahn, Michael A. Skeide, Dante Mantini, Marco Ganzetti, Alain Destexhe, Angela D. Friederici, Gustavo Deco.


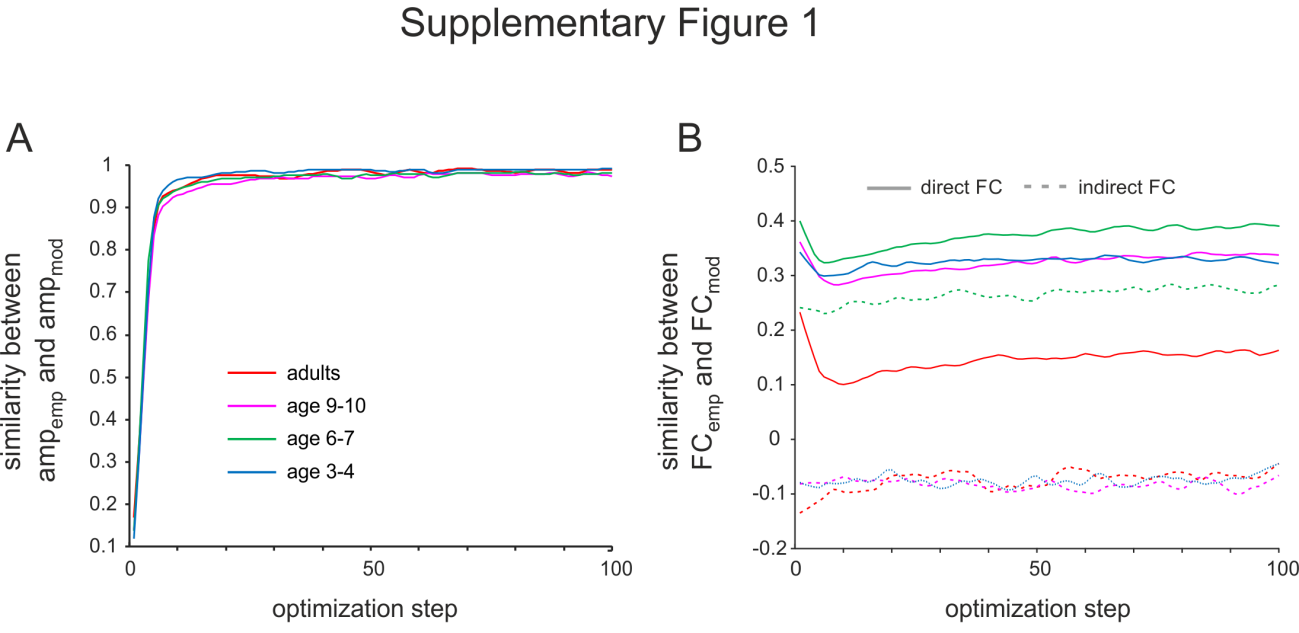


**Supplementary Fig. 1.** Fitting local node dynamics to empirical data (A) Similarity (correlation coefficient) between empirical and simulated power spectrum amplitude obtained for each area as a function of optimization steps and all four age groups. (B) Similarity (correlation coefficient) between empirical and model functional connectivity matrices shown for different levels of model optimization of power spectrum amplitude as depicted in (A) and for direct and indirect FC.


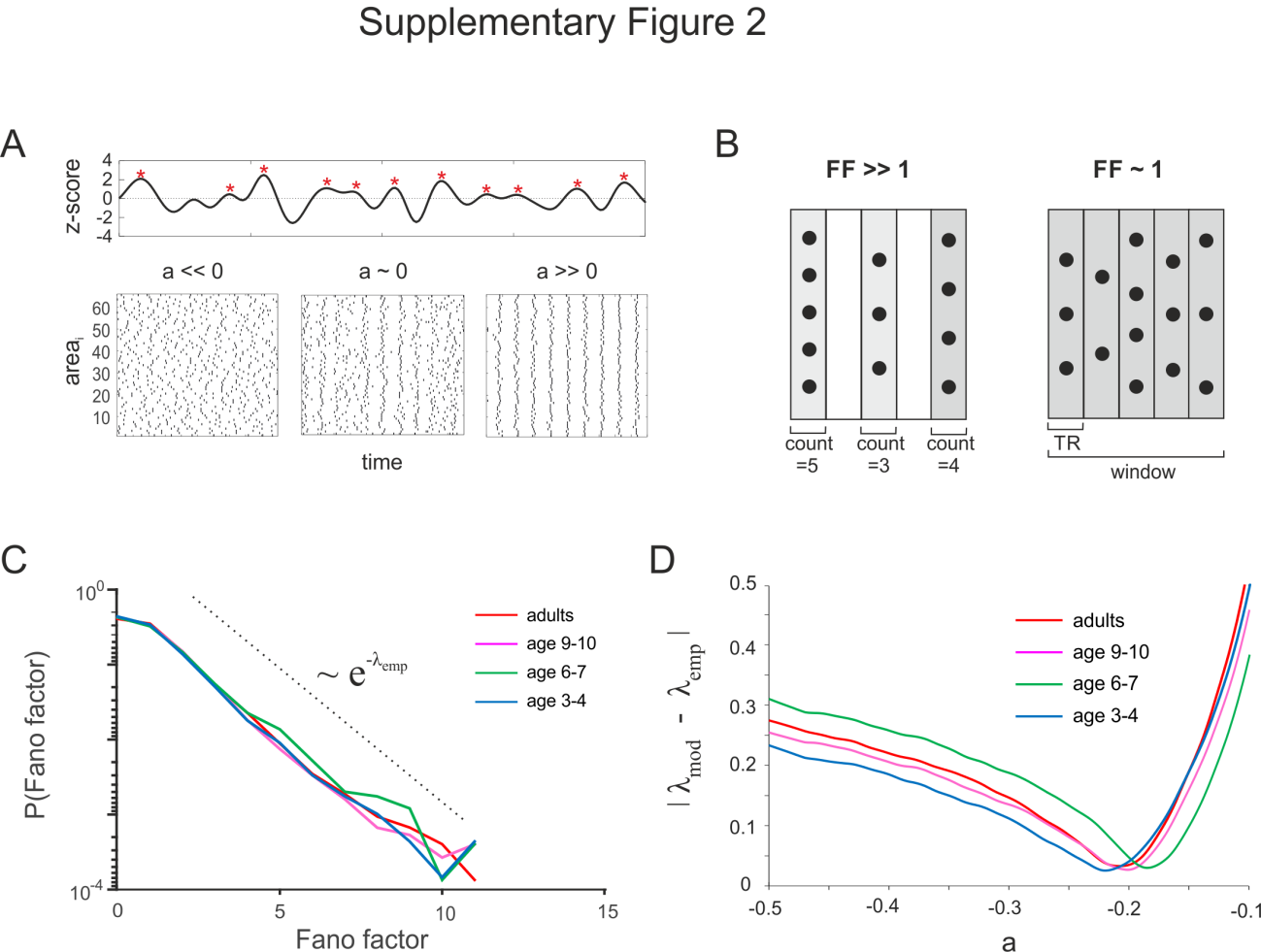


**Supplementary Fig. 2** Analysis of global dynamics (A) Top: Example of an empirical time series and its positive peaks (red asterisk). Bottom: Raster plots for positive peak times (dots) obtained from model time series and connected based on the adult SC matrix for three different values of the bifurcation parameter (a). The bifurcation parameter was uniform across all nodes. (B) Schematic raster plot of peak events for a time window of 5 TR showing different global synchronization levels and associated Fano factors (FF). (C) Empirical Fano factor distributions for all four age groups calculated for window size = 5TR. The distribution follows an exponential distribution with exponent *λ* (dashed line). (D) Absolute difference between empirical and model FF distribution exponent as a function of the bifurcation parameter (a) and all four age groups.


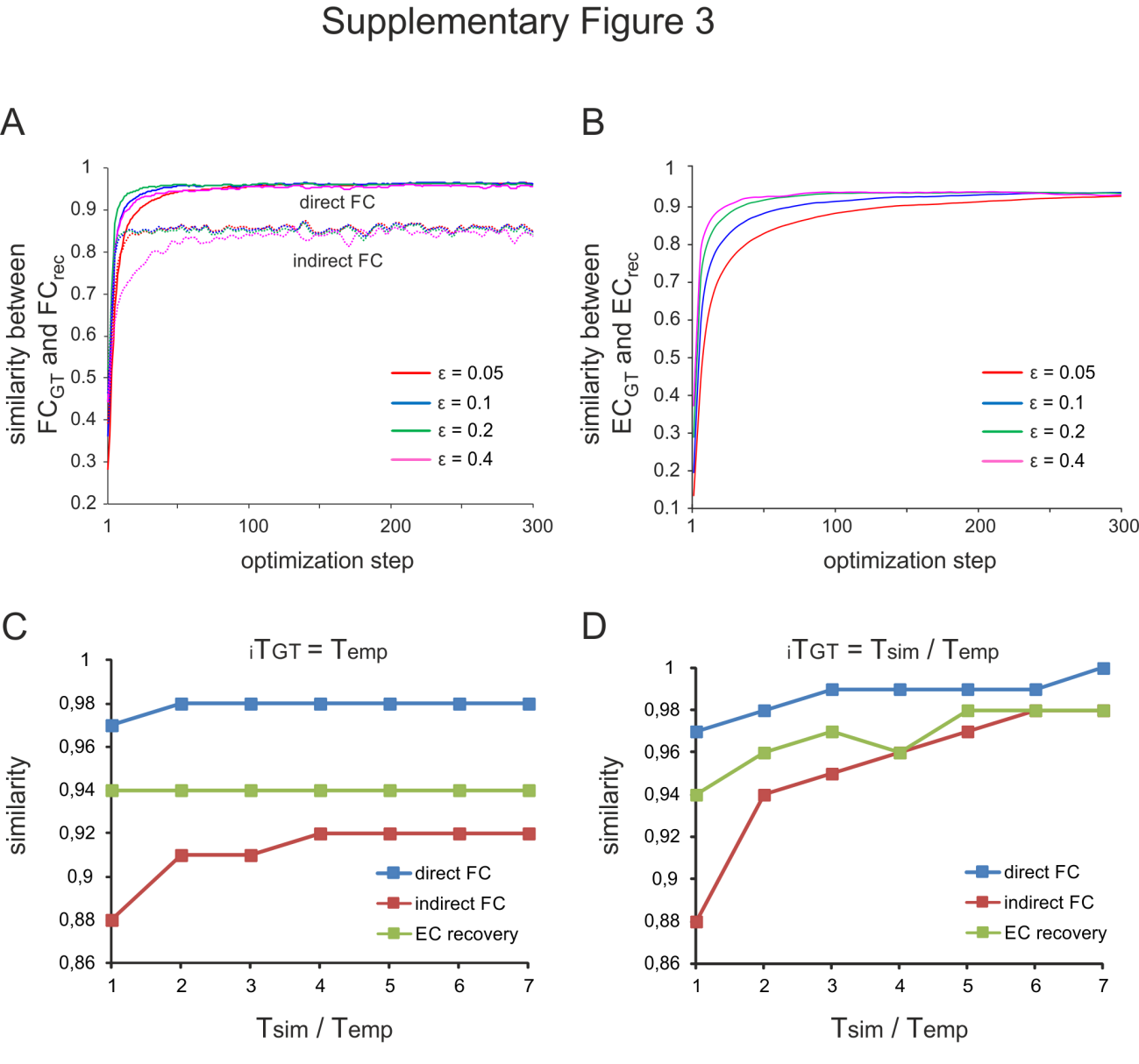


**Supplementary Fig. 3** Model validation with ground truth data. (A) Similarity (Pearson correlation coefficient) between ground truth and recovered model FC for direct and indirect functional connectivity. The FC recovery process is shown for different values of the gradient descent learning parameter ε. (B) Same as in (A), but showing the similarity between the recovered and ground truth EC as a function of gradient descent optimization steps. (C) Similarity between ground truth FC(direct, indirect) – EC, and recovered FC(direct, indirect) – EC, as a function of simulation time with respect to the empirical duration of the dataset. T_sim_/T_emp_ = 1 corresponds to the duration of the empirical recording for an entire age group (T_emp_, 20 subjects) and was used to obtain the ground truth FC. (D) Same as in (C), but the simulation times to obtain the ground truth FC and FC recovery were identical.

**
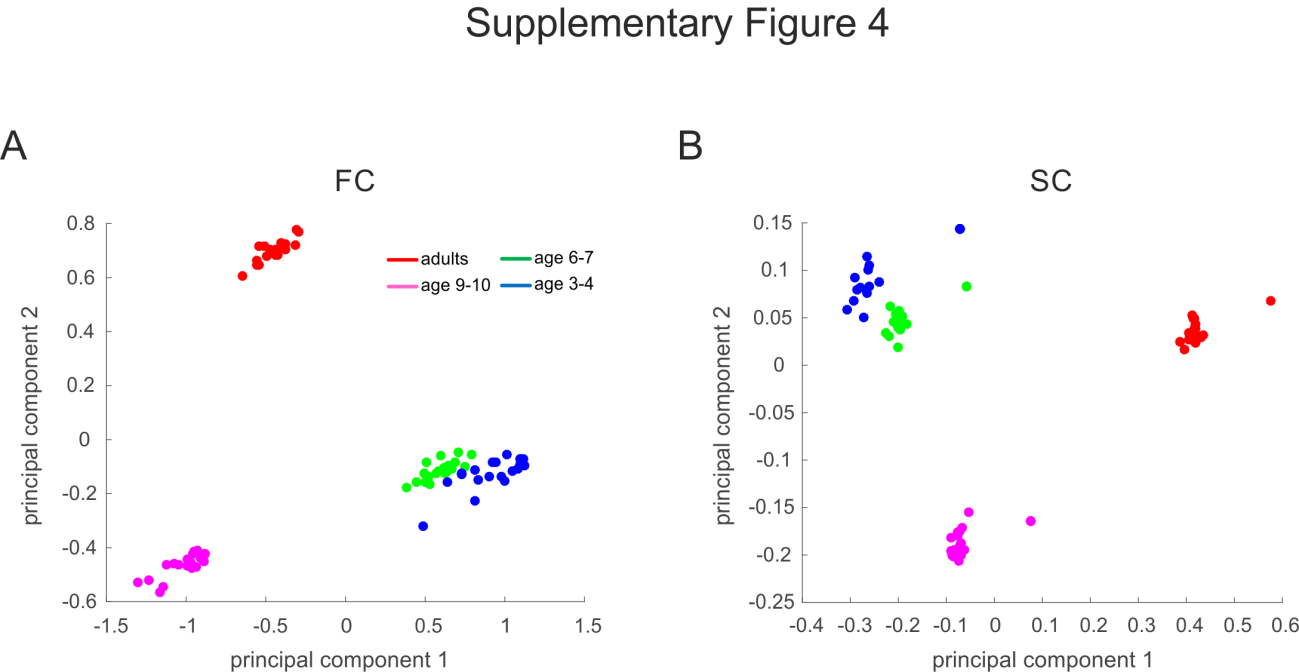
**

**Supplementary Fig. 4** Principal component space of FC and SC. (A) Two- dimensional principal component space of FC node strength across all 66 areas for all age groups. Each data point was obtained by PCA of FC node strengths from all subjects of a group leaving one subject per group out. (B) Same as in (A) for SC.

**Supplementary Discussion**

**Global dynamics**

Changes in the bifurcation parameter not only influenced local dynamics and pairwise interaction strength (see Fig 1A-B in main text), but also had an impact on collective network dynamics and concomitant higher order correlations, i.e. correlations that span more than two areas. Similar effects were seen when the structural matrix was scaled with different values of the global coupling parameter. To illustrate collective dynamical effects we converted the continuous empirical and simulated signals into a point process by applying a threshold (Supplementary Fig. 2A, top; see Materials and Methods). Example raster plots of the discretized model data are shown in Supplementary Fig. 2A (bottom) for fixed structural weights and different values of the bifurcation parameter (a) that were uniform across all nodes. For strongly negative values of the bifurcation parameter, the population dynamics shows no apparent global synchronization, while positive values of the bifurcation parameter and a concomitant transition to sustained local oscillations was followed by strong global coordination of simulated activity across the entire brain. As the bifurcation parameter approached the bifurcation point, the model dynamics displayed random transitions between network-wide synchronization and global desynchronization. To quantify higher order correlations stemming from collective synchronization, we moved a sliding window of five TR across the discretisized data and calculated the Fano factor for each window (Supplementary Fig. 2C and see Materials and Methods). A Fano factor ~ 1 indicated independence between all events within a time window, while FF values >>1 reflected increasing levels of global synchronization across all areas.

To compare higher order interactions between the empirical and model data, we first computed Fano factor distributions for each age group reflecting FF probabilities across all windows of a given dataset (Supplementary Fig. 2C). These distributions were very similar across the four age groups and decayed according to an exponential function with exponent λ (0.031±0.003) and a cut-off at FF~10. Next, we obtained FF distributions from the simulated model data for different values of the bifurcation parameter and the global coupling parameter, and also observed an exponential spread of Fano factors. We found that the exponential distribution became flatter with higher bifurcation parameter and the fitted exponent λ increased. To precisely model the global synchronization pattern present in the data, we systematically varied the bifurcation parameter, and compared the simulated and empirical Fano factor distributions through their values of λ (Supplementary Fig 2D). We found a distinct minimum for each age group with similar values for the bifurcation parameter (adults: -0.2, age 9-10: -0.2, gage 6-7: -0.19, age 3-4: -0.22), indicating that the global dynamical working point of the model did not distinguish between different age groups. During effective connectivity estimation the global working point was kept constant by rescaling the effective connectivity matrix with a constant such that λ in the model was equal to the λ in the data (Materials and Methods).

**Model validation and ground truth analysis**

To validate the model and test whether it can correctly find a-priori known EC we created ground truth data and applied the algorithm with different randomized initial conditions (Materials and Methods) and different simulation times. Application of this approach consistently yielded fits of ~0.95 for direct FC and ~0.85 for indirect FC for a large range of ε (Supplementary Fig. 3A) and for simulation times that matched the duration of the concatenated data of each age group. The recuperation of the EC resulted in a similarly high value of ~0.93 for the tested range of ε (Supplementary Fig. 3B). Importantly, the optimization of the EC was slower and converged to a stable value after more optimization steps than the FC.

Next, we tested the role of simulation time on the performance of the algorithm. To this end we first simulated ground truth data for a duration of the group datasets and systematically increased the simulation time for recovering the original EC. The results clearly show that neither FC nor EC recovery can be improved by extending simulation time of the algorithm (Supplementary Fig. 3C). Then, we increased duration of the ground truth simulation to larger values than the empirical data and used the same duration to recuperate connectivity parameters. This change had a strong effect on FC and EC such that with sufficient simulation time, direct and indirect FC were recoverable up to ~0.99 and ~0.97, respectively (Fig. 3E). The same results were obtained with the EC, which was recuperated with up to ~0.97 accuracy. These findings suggest that data with long duration favor optimal functioning of the algorithm. Even though the duration of the recordings was insufficient to theoretically reach highest reconstruction of EC, the recuperation performance of ground truth data matching the empirical durations was only little below the optimum (see above).
